# In silico CD4+, CD8+ T-cell and B-cell immunity associated immunogenic epitope prediction and HLA distribution analysis of Zika virus

**DOI:** 10.17179/excli2016-719

**Published:** 2017-01-13

**Authors:** Essam Mohammed Janahi, Anupam Dhasmana, Vandana Srivastava, Aditya Narayan Sarangi, Sana Raza, Jamal M. Arif, Madan Lal Bramha Bhatt, Mohtashim Lohani, Mohammed Yahya Areeshi, Anand Murari Saxena, Shafiul Haque

**Affiliations:** 1 Department of Biology, College of Science, University of Bahrain, P.O. Box 32038, Kingdom of Bahrain; 2 Research Cell, Amity University Lucknow Campus, Lucknow-226028, UP, India; 3 Department of Radiotherapy, King George Medical University, Lucknow-226003, UP, India; 4 Department of Zoology, Lucknow University, Lucknow-226007, UP, India; 5 Biomedical Informatics Centre, Sanjay Gandhi Postgraduate Institute of Medical Sciences, Lucknow-226014, UP, India; 6 Department of Biosciences, Integral University, Lucknow-226026, UP, India; 7 Department of Biochemistry, University of Hail, Hail-2440, Saudi Arabia; 8 Research and Scientific Studies Unit, College of Nursing and Allied Health Sciences, Jazan University, Jazan-45142, Saudi Arabia; 9 Department of Biosciences, Jamia Millia Islamia (A Central University), New Delhi-110025, India

**Keywords:** Zika virus, HLA, vaccine, prophylactic, immunogens, B-/T-cell

## Abstract

Zika virus (ZIKV) is a mosquito-borne flavivirus distributed all over Africa, South America and Asia. The infection with the virus may cause acute febrile sickness that clinically resembles dengue fever, yet there is no vaccine, no satisfactory treatment, and no means of evaluating the risk of the disease or prognosis in the infected people. In the present study, the efficacy of the host's immune response in reducing the risk of infectious diseases was taken into account to carry out immuno-informatics driven epitope screening strategy of vaccine candidates against ZIKV. In this study, HLA distribution analysis was done to ensure the coverage of the vast majority of the population. Systematic screening of effective dominant immunogens was done with the help of Immune Epitope & ABCPred databases. The outcomes suggested that the predicted epitopes may be protective immunogens with highly conserved sequences and bear potential to induce both protective neutralizing antibodies, T & B cell responses. A total of 25 CD4+ and 16 CD8+ peptides were screened for T-cell mediated immunity. The predicted epitope "TGLDFSDLYYLTMNNKHWLV" was selected as a highly immunogenic epitope for humoral immunity. These peptides were further screened as non-toxic, immunogenic and non-mutated residues of envelop viral protein. The predicted epitope could work as suitable candidate(s) for peptide based vaccine development. Further, experimental validation of these epitopes is warranted to ensure the potential of B- and T-cells stimulation for their efficient use as vaccine candidates, and as diagnostic agents against ZIKV.

## Abbreviations

ZIKV: Zika virus; NCR: non-coding regions; ORF: open reading frame; MHC: major histocompatibility complex; HLA: human leukocyte antigen

## Introduction

Zika virus (ZIKV), a pathogenic vector borne flavivirus is a member of the Flaviviridae family, transmitted by the Aedes mosquitoes and is known to cause Zika fever. This virus was first identified in the blood of a sentinel Rhesus monkey during a study on Yellow fever in the Zika forest of Uganda in 1947. This was followed by subsequent isolation of this virus from a pool of Aedes mosquitoes of African region (Dick et al., 1952[9]). Since then, several outbreaks of Zika have been reported in humans in Africa and Asia. Currently, it has been reported in Brazil, the Pacific region, and is also known to spread widely in Central and South America. 

The Zika fever illness is generally characterized by some of the symptoms which are similar to other arbovirus infections like Dengue and Chikungunya. These symptoms include fever, malaise, conjunctivitis, rashes on skin, anorexia, headache as well as pain in the muscles and joints (Simpson, 1964[19]). In case of pregnant women, Zika virus may spread from the mother to the fetus and is believed to be associated with microcephaly, a serious birth defect characterized by smaller and poorly developed brain in the newborns (Torjesen, 2016[20]). Pregnant women are therefore advised to avoid traveling to areas prone to incidence of Zika. Zika virus has 25-30 nm nucleocapsid, which is surrounded by a lipid bilayer containing envelope proteins. It has a single stranded RNA genome with 10,141 base pairs accountable for encoding of nearly 3380 amino acids (Fonseca et al., 2014[12]). The virus has two flanking 5' and 3' non-coding regions (NCR) and a long open reading frame (ORF) “59-C-prM-E-NS1-NS2A-NS2BNS3-NS4A-NS4B-NS5-39” coding for a polyprotein that cleaves into its envelope (E), capsid (C), precursor membrane (prM), and some other non-structural (NS) proteins (Chambers et al., 1990[5]; Kuno et al., 2007[14]). Zika virus is maintained through a sylvatic transmission cycle. The envelope protein is the major surface protein of the virus, and is involved in various aspects of the viral cycle and is believed to mediate binding and membrane fusion (Lindenbach et al., 2003[15]). The non-structural protein, NS5 has RNA-dependent RNA polymerase (RdRP) that acts at its C-terminal, while the N-terminal is involved in RNA capping (Lindenbach and Rice, 2003[15]). The 3' non-coding region is made up of about 428 nucleotides having 27 folding patterns (Kuno et al., 2007[14]), which are believed to be involved in the processes like translation, stabilization of the genome, packaging of RNA, and recognition of the cellular or the viral factors (Lindenbach and Rice, 2003[15]). 

Due to lack of rapid diagnostic techniques, effective treatment and availability of novel vaccines against the Zika virus has been a public health emergency of the international concern declared by the World Health Organization (Salaam-Blyther, 2016[17]). Hence, it is necessary to develop a substantial amount of immunity in humans against Zika virus to prevent the widespread outbreak of the infection and its associated effects. Taking a step towards this development, we screened the immunogenic epitopes of the envelope protein of Zika virus using an *in silico* approach. This might be helpful in developing peptide based vaccines against the Zika virus.

## Materials and Methods

### Sequence retrieval

The 504 length amino acid sequence of viral envelope (E) protein (GenBank: AIC06934.1 & RCSB PDB ID: 5IZ7), involved in the host cell binding and fusion activity was retrieved from UniprotKB Database (www.uniprot.org). The retrieved sequence (A0A060H177_9FLAV E protein) was a further subjected to immunogenicity assessment and epitope prediction. 

### Immunogenicity prediction of the viral protein

The VaxiJen V2.0 server (http://www.ddg-pharmfac.net/vaxijen/VaxiJen/VaxiJen.html) was used for the evaluation of immunogenicity of the retrieved protein sequence. This server works on Auto Cross Covariance (ACC) algorithm that predicts protective antigens, tumor antigens, and subunit vaccines with the precision level of 70 to 89 % for the discrimination between antigens and non-antigens (Doytchinova and Flower, 2007[10]).

### Epitope prediction

#### (a) B-cell epitope prediction

B-cell epitopes were predicted using an ABCPred online server (www.imtech.res.in/abcpred) by selecting the window length of 20 amino acids, which were recommended for humaoral immunity. This server is based on information - processing algorithms inspired by the biological nervous system. The server ranks the epitopes as per their respective scores. The higher score of the peptides means a greater probability of becoming most suitable immunogenic epitope (Saha et al., 2006[16]).

#### (b) CD4+ and CD8+ epitope prediction

The envelop protein sequence was analyzed for the screening of the possible dominant T-cell epitopes using immuno-informatics (Immune Epitope Database) tool IEDB online server (www.iedb.org) (Bui et al., 2006[4]). Human leukocyte antigen (HLA)-I and HLA-II binding resources present in the IEDB, were used for the binding analysis of all the possible peptides considering the presence of all the HLAs in the database. The peptide length was set to be 10 and 15 for HLA-I and HLA-II, respectively. Default IEDB recommended prediction method combines the predictions from ANN, SMM, and CombLib algorithms. The epitopes were predicted on the basis of lowest percentile rank and high binding affinity.

### Antigenicity of the identified epitopes

The immunogenicity of both B-cell and T-cell predicted epitopes was evaluated using a VexiJen V2.0 online server (http://www.ddg-pharmfac.net/vaxijen/VaxiJen/VaxiJen.html)

### Determination of conserved regions & toxicity of the identified epitopes

The immunogenic epitopes were checked for the conserved regions and further subjected to ToxinPred severe (Gupta et al., 2013[13]) for the differentiation of toxic or non-toxic peptides. The ToxinPred server is based on Support Vector Machine (SVM) and Quantitative Matrix based algorithm, and generates quantitative matrix on the basis of probability or frequency of amino acid at a particular position. 

### Determination of physico-chemical properties of the identified epitopes

The Peptide Property Calculator (https://www.genscript.com) server was used to determine the best solvent for the predicted peptide based on its amino acid sequence.

### Population coverage analysis

**Figure 1:**
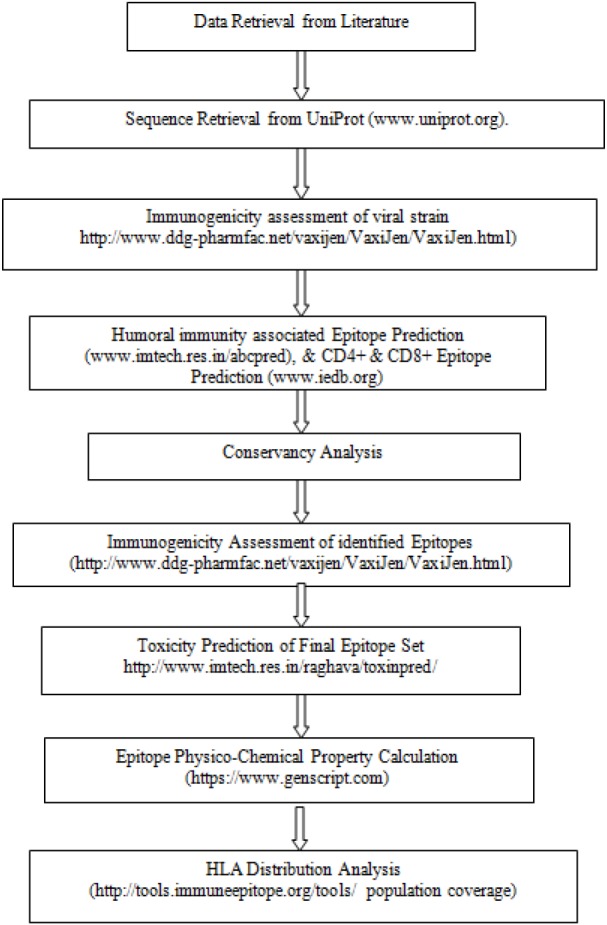
Schematic representation of the entire *in silico* strategy of CD4+, CD8+ T-cell and B-cell epitope prediction and HLA distribution of Zika virus

Due to the dependence of major histocompatibility complex (MHC) of T-cell response, the peptides with wide range of HLA binding specificities result in wider population coverage in terms of geographical expansion. The population coverage rate of the predicted epitopes was calculated by employing the IEDB population coverage tool (http://tools.immuneepitope.org/tools/population/iedb_input)(Bui et al., 2006[4]). The predicted epitopes with its all binding HLA alleles for the worldwide distribution were tabulated. The schematic representation of the entire methodology of *in silico* CD4+, CD8+ T-cell & B-cell epitope prediction and HLA distribution of Zika virus is given in Figure 1(Fig. 1).

## Results

### Protein immunogenicity and epitope identification

The immunogenicity of the viral envelope protein was ensured using VaxiJen V2.0 online server, keeping the threshold at 0.4. The results obtained suggest that the viral sequence is a probable antigen with a score of 0.6178. The prediction and identification of B-cell epitopes in target antigens, being a key step in the epitope based vaccine development, was performed by using ABCPred server. Based on the artificial neural network (ANN) method, the epitopes predicted by ABCPred were in the descending order of their scores, depicting that the top most has the best binding affinity. Therefore, the first (top) epitope 'TGLDFSDLYYLTMNNKHWLV' with the score 0.90 at the position 194 to 213 showing the highest probability to be an antigenic determinant, was selected for further study. 

MHC-II restricted CD4+ T-cells activation plays an important role in initiating and upholding a proficient antibody response or cytotoxic T-lymphocytes (CTL) response. Further, MHC-I restricted CD8+ CTLs have a vital role in combating the viral infection. Thus, the comprehending principles of T-cell activation and epitope-based vaccine design, direct the identification of helper T-cell epitopes and CTL epitopes. The helper T-cell epitopes and CTL epitopes in the 'Env' protein of Zika virus were identified using the IEDB online tool. The immunogenic peptides were ranked in accordance with their consensus percentile rank from SWISS-PROT program. The lowest percentile rank demonstrated as the best binders. By selecting the first (lowest percentile rank) epitope for each of HLA class-II and HLA class-I gave 63 helper T-cell epitopes (Supplementary Information: Table S1) and 76 CTL epitopes (Supplementary Information: Table S2).

### Conserved regions and immunogenicity of the identified epitopes

Peptides with strong antigenicity are always better candidates to be B- and T-cell epitopes than those with weak antigenicity. Hence, the assessment of immunogenicity of the identified peptides was performed using the VaxiJen V2.0 server (http://www.ddg-pharmfac.net/vaxijen/VaxiJen/VaxiJen.html). This tool predicts the antigenicity of a peptide on the basis of amino acid properties and their positions in the peptide. The results demonstrated that the identified B-cell epitope is immunogenic in nature with score of 1.2758, and among all the identified T-cell epitopes, a total of 16 HLA class-II and 25 HLA class-I epitopes were found to be conserved as well as immunogenic (Supplementary Information: Table S3). 

### Toxicity and solvency analysis

The final epitope set (B- and T-cell epitopes) was further tested for the toxicity analysis and medium solvency. All the predicted immunogenic epitopes were non-toxic and were classified as acidic, basic or neutral (Table 1(Tab. 1)). Acidic peptides were initially dissolved in water, if not, then < 50 µl NH_4_OH was added and diluted to 1 ml with deionized water. For basic peptides, 10 % and higher solutions of acetic acid was tried; in case not dissolved, then < 50 µl TFA was added and diluted up to 1 ml with deionized water to solubilize the peptide. Neutral peptides required organic solvents like acetonitrile, methanol, or isopropanol.

### Population coverage analysis

HLA allocation varies among diverse ethnic groups spread over different geographical regions of the world. Therefore, while designing a potent vaccine, population coverage must be taken into account to cover maximum possible populations. In this study, all alleles in IEDB database were identified as best binders with the predicted epitopes and were used to establish the population coverage for these epitopes (Table 2(Tab. 2)). A significant percentage of the specific area population coverage of all the identified immunogenic epitopes was found all over the world. 

## Discussion

Vaccination appears to be the most optimal and successful strategy to reduce the menace of dreadful infectious diseases worldwide. Currently, the immune-informatics approach is the most promising approach for the development of peptide based vaccines. Due to lack of effective treatment for the microcephaly caused by Zika virus, there is an urgent need for a cost effective vaccine against this deadly virus. Earlier studies have already demonstrated that application of potential epitope(s) instead of whole protein as a vaccine has been confirmed to represent the complete antigenicity of any protein (DeGroot et al., 2002[8]). Also, in the recent past, the epitope based vaccines have given promising results against highly infectious diseases such as H1N1, HIV and Tuberculosis (Correia et al., 2014[6]). These vaccines may have a lower chance of eliciting immune response against self-antigens, thereby avoiding autoimmune responses (Aziz et al., 2008[2]). The traditional molecular immunology techniques used to search for immunogenic peptides to be used as vaccine candidates are extensively time consuming, labour intensive, and expensive. However, as a remedy, in recent years, various computational approaches have been proposed and tested by many researchers to reduce the time, cost, and labour involved (Davies and Flower, 2007[7]; Shi et al., 2015[18]). On the similar lines, the suggested *in silico *strategy might prove as a boon for the experimental biologists in rapid screening and identification of probable vaccine candidates. T-lymphocytes play a central role in the generation of a protective immune response in many microbial infections (Esser et al., 2003[11]), hence the prediction and screening of T-cell epitopes can be exploited as a potential vaccine candidate is a critical requirement for the designing of epitope-based vaccines.

Recently, a similar approach was used for the identification of highly immunogenic peptides of Env protein of Zika virus (Badawi et al., 2016[3]) and suggested highly recommended T cell epitopes MMLELDPPF as therapeutic peptide vaccine to interact with MHC class I along with three other epitopes MAVLGDTAW, KEWFHDIPL and DTAWDFGSV. Also, three epitopes showed high affinity to interact with MHC class II alleles i.e., FKSLFGGMS, LITANPVIT and VHTALAGAL. Alam et al. (2016[1]) proposed MHC class-I potential peptides YRIMLSVHG, VLIFLSTAV and MMLELDPPF, GLDFSDLYY as potent peptide epitopes for CD4+ and CD8+ T-cells, respectively. The current study was aimed to identify B-cell, T-cell highly immunogenic, non toxic and non variable peptides which have maximum world population coverage area.

The sequence of the viral envelope protein was used for the evaluation of the viral immunogenicity to predict the vaccine candidates against the immune-responses of B- and T-cells. The B-cell epitope and the best/highest percentile score generated a single epitope 'TGLDFSDLYYLTMNNKHWLV' at a position of 194 to 213 with immunogenic score 1.2758. The putative CD4+ & CD8+ T-cell core epitopic sequences for all the HLA super types were predicted. We also checked our predicted immunogenic epitopes for the conserved regions and further subjected them to ToxinPred server for the differentiation of toxic or non-toxic peptides. The assessment of the immunogenicity and toxicity properties of the putative epitopes using various online servers showed that the predicted epitopes were immunogenic in nature, and no sequence variability was detected in the region containing the potential epitopes.

The epitope NTKNGSISLMCLALG at position 478-492 for HLA Class-II and the epitope RLSSGHLKCR at position 283-292 for HLA Class-I was found to have the highest antigenic score of 1.9904 and 2.0901, respectively, and both were found to be basic in nature (Table 1(Tab. 1)). 

The putative epitopes have excellent worldwide population coverage as depicted in Table 2(Tab. 2), and may provide worldwide immune protection (83.71 % of the average MHC Class-I coverage and 96.37 % of the average MHC Class-II coverage) against ZIKV. 

The application of *in silico* approaches by immunologists has helped to speed up the *in vitro, in vivo* and *ex vivo* studies by cutting down the large epitope dataset into a smaller one that needs to be confirmed by the experimental analysis. The predictions made by the present study on the primary amino acid sequence of Zika virus should help other researchers to experimentally verify the immuno-dominant epitopes fundamental to the understanding of the pathobiology of Zika virus leading to effective vaccine development.

The data from the current work on B- and T-cell peptide epitopes for 'Env' protein of Zika virus might prove a milestone in the development of vaccines to combat Zika virus induced infection. The suggested *in silico *strategy might prove as a boon for the experimental biologists in rapid screening and identification of probable vaccine candidates. The proposed non-toxic epitopes would be a relevant representative of a large proportion of the human population. This work indicates that the proposed epitopes prompt the future vaccine development against the Zika virus. However, the potential of the screened epitopes by experimental validation is warranted.

## Notes

Essam Mohammed Janahia and Shafiul Haque (Research and Scientific Studies Unit, College of Nursing and Allied Health Sciences, Jazan University, Jazan-45142, Saudi Arabia; Phone & Fax No.: +966-1-73174383, E-mail: shafiul.haque@hotmail.com) contributed equally as corresponding authors.

## Supplementary Information

**Table S1. **Predicted immunogenic peptides for the HLA class-II

**Table S2. **Predicted immunogenic peptides for the HLA class-I

**Table S3. **Immunogenicity assessment of the identified peptides using the VaxiJen

## Statement of conflicts of interest

The authors declare no conflicts of interest.

## Acknowledgements

The authors are grateful to Amity University Lucknow Campus, UP, India, for providing the necessary facilities for this study. Also, the authors thank the Indian Council of Medical Research, New Delhi, India, for providing Research Associateship to Dr. Aupam Dhasmana (File No- 45/20/2013/NAN-BMS).

## Supplementary Material

Supplementary information

## Figures and Tables

**Table 1:**
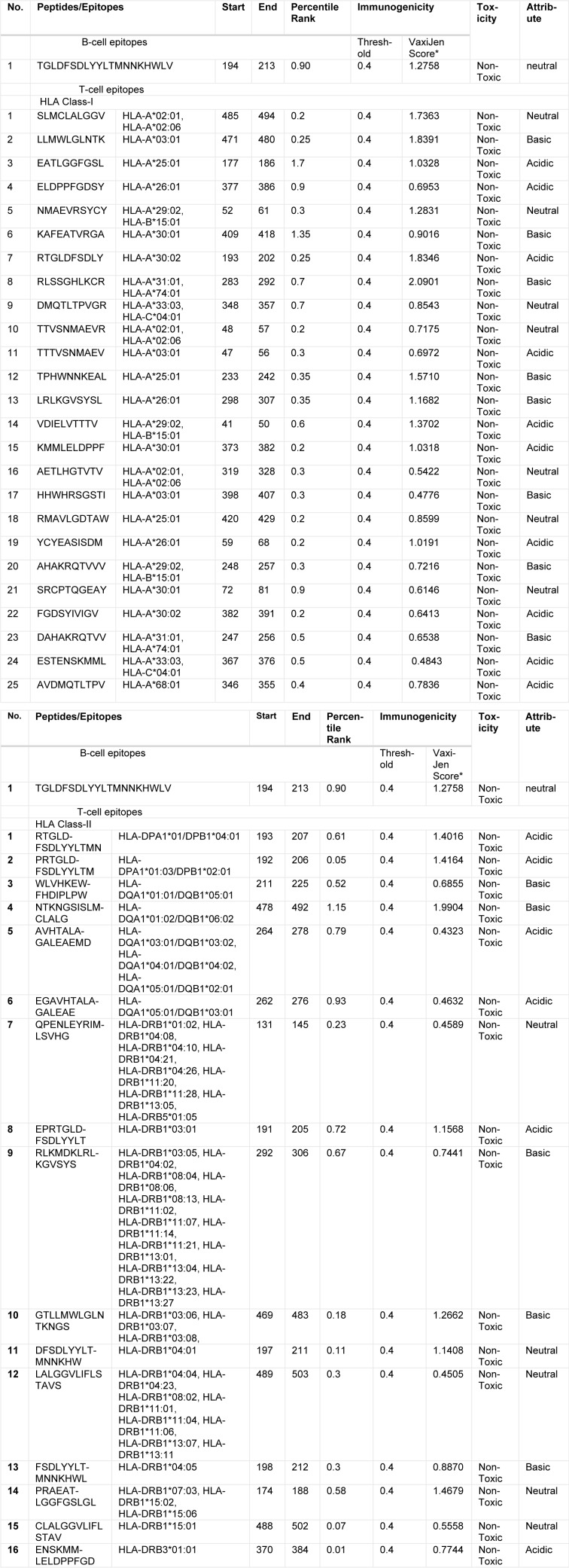
Prediction of most immunogenic, non-mutant and non-toxic B-cell and T-cell epitopes of the envelope protein of Zika virus (the threshold immunogenicity of the peptides was 0.4 and the total predicted range of VaxiJen score was from 2.0901 to 0.4505)

**Table 2:**
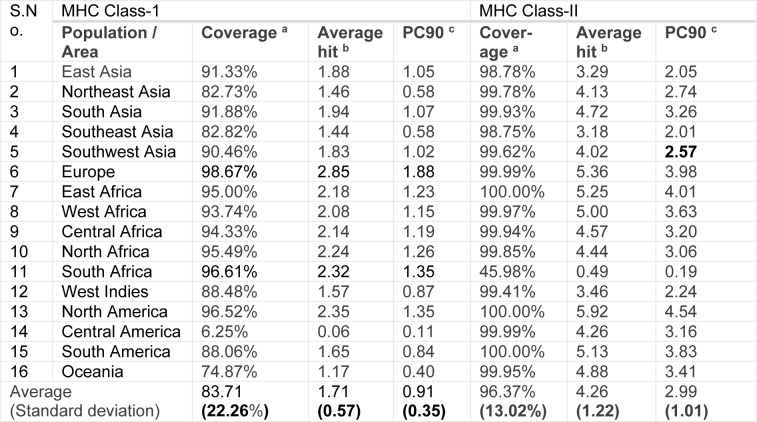
Prediction of population coverage rate (%) of MHC Class-I and MHC Class-II

## References

[1] Alam A, Ali S, Ahmad S, Malik MZ, Ishrat R (2016). From ZikV genome to vaccine: in silico approach for the epitope-based peptide vaccine against Zika virus envelope glycoprotein. Immunology.

[2] Azizi A, Anderson DE, Torres JV, Ogrel A, Ghorbani M, Soare C (2008). Induction of a broad cross-subtype-specific HIV-1 immune responses by a novel multivalent HIV-1 peptide vaccine in cynomolgus macaques. J Immunol.

[3] Badawi MM, Osman MM, Alla AAF, Ahmedani AM, Abdalla MH, Gasemelseed MM (2016). Highly conserved epitopes of ZIKA envelope glycoprotein may act as a novel peptide vaccine with high coverage: Immunoinformatics approach. Am J Biomed Res.

[4] Bui HH, Sidney J, Dinh K, Southwood S, Newman MJ, Sette A (2006). Predicting population coverage of T-cell epitope-based diagnostics and vaccines. BMC Bioinformatics.

[5] Chambers TJ, Hahn CS, Galler R, Rice CM (1990). Flavivirus genome organization, expression, and replication. Annu Rev Microbiol.

[6] Correia BE, Bates JT, Loomis RJ, Baneyx G, Carrico C, Jardine JG (2014). Proof of principle for epitope-focused vaccine design. Nature.

[7] Davies MN, Flower DR (2007). Harnessing bioinformatics to discover new vaccines. Drug Discov Today.

[8] DeGroot AS, Sbai H, Aubin CS, McMurry J, Martin W (2002). Immuno-informatics: Mining genomes for vaccine components. Immunol Cell Biol.

[9] Dick GW, Kitchen SF, Haddow AJ (1952). Zika virus isolations and serological specificity. Trans R Soc Trop Med Hyg.

[10] Doytchinova IA, Flower DR (2007). VaxiJen: a server for prediction of protective antigens, tumour antigens and subunit vaccines. BMC Bioinformatics.

[11] Esser MT, Marchese RD, Kierstead LS, Tussey LG, Wang F, Chirmule N (2003). Memory T cells and vaccines. Vaccine.

[12] Fonseca K, Meatherall B, Zarra D, Drebot M, MacDonald J, Pabbaraju K (2014). First case of zika virus infection in a returning canadian traveller. Am J Trop Med Hyg.

[13] Gupta S, Kapoor P, Chaudhary K, Gautam A, Kumar R (2013). In silico approach for predicting toxicity of peptides and proteins. PLoS ONE.

[14] Kuno G, Chang JJ (2007). Full-length sequencing and genomic characterization of Bagaza, Kedougou, and Zika viruses. Arch Virol.

[15] Lindenbach BD, Rice CM (2003). Molecular biology of flaviviruses. Adv Virus Res.

[16] Saha S, Raghava GPS (2006). Prediction of continuous B-cell epitopes in an antigen using recurrent neural network. Proteins.

[17] Salaam-Blyther T (2016). Zika virus: global health considerations. CRS INSIGHT.

[18] Shi J, Zhang J, Li S, Sun J, Teng Y, Wu M (2015). Epitope-based vaccine target screening against highly pathogenic MERS-CoV: An in silico approach applied to emerging infectious diseases. PLoS ONE.

[19] Simpson DI (1964). Zika virus infection in man. Trans R Soc Trop Med Hyg.

[20] Torjesen I (2016). Zika virus outbreaks prompt warnings to pregnant women. BMJ.

