# Supplementary material for: In silico CD4+, CD8+ T-cell and B-cell immunity associated immunogenic epitope prediction and HLA distribution analysis of Zika virus
Source: EXCLI J. 2017 Jan 13;16:63–72. doi: 10.17179/excli2016-719 (PMC5379118; doi:10.17179/excli2016-719)
Supplement: Supplementary information [file EXCLI-16-63-s-001.pdf]

EXCLI Journal 2017;16:63-72 – ISSN 1611-2156
Received: October 20, 2016, accepted: January 02, 2017, published: January 13, 2017

Supplementary information to:

IN SILICO CD4+, CD8+ T-CELL AND B-CELL IMMUNITY ASSOCIATED IMMUNOGENIC EPITOPE PREDICTION AND HLA DISTRIBUTION ANALYSIS OF ZIKA VIRUS

Essam Mohammed Janahi[a*], Anupam Dhasmana[b,c], Vandana Srivastava[d], Aditya Narayan Sarangi[e], Sana Raza[c,f], Jamal M. Arif[g], Madan Lal Bramha Bhatt[c], Mohtashim Lohani[c,h], Mohammed Yahya Areeshi[h], Anand Murari Saxena[d], Shafiul Haque[h,i]*

[a] Department of Biology, College of Science, University of Bahrain, P.O. Box 32038, Kingdom of Bahrain
[b] Research Cell, Amity University Lucknow Campus, Lucknow-226028, UP, India
[c] Department of Radiotherapy, King George Medical University, Lucknow-226003, UP, India
[d] Department of Zoology, Lucknow University, Lucknow-226007, UP, India
[e] Biomedical Informatics Centre, Sanjay Gandhi Postgraduate Institute of Medical Sciences, Lucknow-226014, UP, India
[f] Department of Biosciences, Integral University, Lucknow-226026, UP, India
[g] Department of Biochemistry, University of Hail, Hail-2440, Saudi Arabia
[h] Research and Scientific Studies Unit, College of Nursing and Allied Health Sciences, Jazan University, Jazan-45142, Saudi Arabia
[i] Department of Biosciences, Jamia Millia Islamia (A Central University), New Delhi-110025, India
* Corresponding authors: Dr. Shafiul Haque, Research and Scientific Studies Unit, College of Nursing and Allied Health Sciences, Jazan University, Jazan-45142, Saudi Arabia, Phone & Fax No.: +966-1-73174383, E-mail: shafiul.haque@hotmail.com
Dr. Essam Mohammed Janahi, Department of Biology, College of Science, University of Bahrain, P.O. Box 32038, Kingdom of Bahrain, Phone: +973-17437425, Fax: +973-1744 9158, Email: emohammed@uob.edu.bh

http://dx.doi.org/10.17179/excli2016-719

This is an Open Access article distributed under the terms of the Creative Commons Attribution License (http://creativecommons.org/licenses/by/4.0/).

EXCLI Journal 2017;16:63-72 – ISSN 1611-2156
Received: October 20, 2016, accepted: January 02, 2017, published: January 13, 2017

Table S1: Predicted immunogenic peptides for HLA Class-II

S.No.	Allele	Start	End	Peptide/Epitopes	Percentile Rank
	**MHC-II**				
**DP Type**					
1	HLA-DPA1*01/DPB1*04:01	193	207	RTGLDFSDLYYLTMN	0.61
2	HLA-DPA1*01:03/DPB1*02:01	192	206	PRTGLDFSDLYYLTM	0.05
3	HLA-DPA1*02:01/DPB1*01:01	460	474	MSWFSQILIGTLLMW	1.54
4	HLA-DPA1*02:01/DPB1*05:01	443	457	KGIHQIFGAAFKSLF	6.57
5	HLA-DPA1*03:01/DPB1*04:02	462	476	WFSQILIGTLLMWLG	1.1
**DQ Type**					
1	HLA-DQA1*01:01/DQB1*05:01	211	225	WLVHKEWFHDIPLPW	0.52
2	HLA-DQA1*01:02/DQB1*06:02	478	492	NTKNGSISLMCLALG	1.15
3	HLA-DQA1*03:01/DQB1*03:02	264	278	AVHTALAGALEAEMD	0.79
4	HLA-DQA1*04:01/DQB1*04:02	264	278	AVHTALAGALEAEMD	0.64
5	HLA-DQA1*05:01/DQB1*02:01	264	278	AVHTALAGALEAEMD	2.16
6	HLA-DQA1*05:01/DQB1*03:01	262	276	EGAVHTALAGALEAE	0.93
**DR Type**					
1	HLA-DRB1*01:01	309	323	TAAFTFTKIPAETLH	1.58
2	HLA-DRB1*01:02	131	145	QPENLEYRIMLSVHG	0.23
3	HLA-DRB1*03:01	191	205	EPRTGLDFSDLYYLT	0.72
4	HLA-DRB1*03:05	292	306	RLKMDKLRLKGVSYS	0.67
5	HLA-DRB1*03:06	469	483	GTLLMWLGLNTKNGS	0.18
6	HLA-DRB1*03:07	469	483	GTLLMWLGLNTKNGS	0.18
7	HLA-DRB1*03:08	469	483	GTLLMWLGLNTKNGS	0.18
8	HLA-DRB1*03:11	489	303	LKCRLKMDKLRLKGV	0.05
9	HLA-DRB1*04:01	197	211	DFSDLYYLTMNNKHW	0.11
10	HLA-DRB1*04:02	292	306	RLKMDKLRLKGVSYS	0.17
	**MHC-II**				
11	HLA-DRB1*04:04	489	503	LALGGVLIFLSTAVS	0.3
12	HLA-DRB1*04:05	198	212	FSDLYYLTMNNKHWL	0.3
13	HLA-DRB1*04:08	131	145	QPENLEYRIMLSVHG	0.03
14	HLA-DRB1*04:10	131	145	QPENLEYRIMLSVHG	0.07
15	HLA-DRB1*04:21	131	145	QPENLEYRIMLSVHG	0.02
16	HLA-DRB1*04:23	489	503	LALGGVLIFLSTAVS	0.03
17	HLA-DRB1*04:26	131	145	QPENLEYRIMLSVHG	0.09
18	HLA-DRB1*07:01	490	504	ALGGVLIFLSTAVSA	0.71

EXCLI Journal 2017;16:63-72 – ISSN 1611-2156
Received: October 20, 2016, accepted: January 02, 2017, published: January 13, 2017

S.No.	Allele	Start	End	Peptide/Epitopes	Percentile Rank
	MHC-II				
19	HLA-DRB1*07:03	174	188	PRAEATLGGFGSLGL	0.58
20	HLA-DRB1*08:01	84	98	KQSDTQYVCKRTLVD	0.01
21	HLA-DRB1*08:02	489	503	LALGGVLIFLSTAVS	0.42
22	HLA-DRB1*08:04	292	306	RLKMDKLRLKGVSYS	0.01
23	HLA-DRB1*08:06	292	306	RLKMDKLRLKGVSYS	0.03
24	HLA-DRB1*08:13	292	306	RLKMDKLRLKGVSYS	0.01
25	HLA-DRB1*09:01	308	322	CTAAFTFTKIPAETL	0.46
26	HLA-DRB1*11:01	489	503	LALGGVLIFLSTAVS	0.34
27	HLA-DRB1*11:02	292	306	RLKMDKLRLKGVSYS	0.02
28	HLA-DRB1*11:04	489	503	LALGGVLIFLSTAVS	0.16
29	HLA-DRB1*11:06	489	503	LALGGVLIFLSTAVS	0.16
30	HLA-DRB1*11:07	292	306	RLKMDKLRLKGVSYS	0.29
31	HLA-DRB1*11:14	292	306	RLKMDKLRLKGVSYS	0.09
32	HLA-DRB1*11:20	131	145	QPENLEYRIMLSVHG	0.08
33	HLA-DRB1*11:21	292	306	RLKMDKLRLKGVSYS	0.02
34	HLA-DRB1*11:28	131	145	QPENLEYRIMLSVHG	0.25
35	HLA-DRB1*12:01	490	504	ALGGVLIFLSTAVSA	0.9
36	HLA-DRB1*13:01	292	306	RLKMDKLRLKGVSYS	0.04
37	HLA-DRB1*13:02	354	368	PVGRLITANPVITES	3.21
38	HLA-DRB1*13:04	292	306	RLKMDKLRLKGVSYS	0.15
39	HLA-DRB1*13:05	131	145	QPENLEYRIMLSVHG	0.25
40	HLA-DRB1*13:07	489	503	LALGGVLIFLSTAVS	0.06
41	HLA-DRB1*13:11	489	503	LALGGVLIFLSTAVS	0.16
42	HLA-DRB1*13:21	84	98	KQSDTQYVCKRTLVD	0.15
43	HLA-DRB1*13:22	292	306	RLKMDKLRLKGVSYS	0.02
44	HLA-DRB1*13:23	292	306	RLKMDKLRLKGVSYS	0.09
45	HLA-DRB1*13:27	292	306	RLKMDKLRLKGVSYS	0.04
46	HLA-DRB1*15:01	488	502	CLALGGVLIFLSTAV	0.07
47	HLA-DRB1*15:02	174	188	PRAEATLGGFGSLGL	0.02
48	HLA-DRB1*15:06	174	188	PRAEATLGGFGSLGL	0.06
49	HLA-DRB3*01:01	370	384	ENSKMMLELDPPFGD	0.01
50	HLA-DRB4*01:01	290	304	KCRLKMDKLRLKGVS	2.76
51	HLA-DRB5*01:01	450	464	GAAFKSLFGGMSWFS	0.31
52	HLA-DRB5*01:05	131	145	QPENLEYRIMLSVHG	0.17

EXCLI Journal 2017;16:63-72 – ISSN 1611-2156
Received: October 20, 2016, accepted: January 02, 2017, published: January 13, 2017

Table S2: Predicted immunogenic peptides for HLA Class-I

S.No.	Allele	Start	End	Peptide/Epitopes	Percentile Rank
**A Type**	**MHC-I**				
1	HLA- A*01:01	81	90	YLDKQSDTQY	0.25
2	HLA-A*02:01	485	494	SLMCLALGGV	0.2
3	HLA-A*02:06	485	494	SLMCLALGGV	0.25
4	HLA-A*03:01	471	480	LLMWLGLNTK	0.25
5	HLA-A*11:01	114	123	VTCAKFACSK	0.45
6	HLA-A*23:01	448	457	IFGAAFKSLF	0.3
7	HLA-A*24:02	448	457	IFGAAFKSLF	0.45
8	HLA-A*25:01	177	186	EATLGGFGSL	1.7
9	HLA-A*26:01	377	386	ELDPPFGDSY	0.9
10	HLA-A*29:02	52	61	NMAEVRSYCY	0.3
11	HLA-A*30:01	409	418	KAFEATVRGA	1.35
12	HLA-A*30:02	193	202	RTGLDFSDLY	0.25
13	HLA-A*31:01	283	292	RLSSGHLKCR	0.7
14	HLA-A*32:01	305	314	YSLCTAAFTF	1.0
15	HLA-A*33:03	348	357	DMQTLTPVGR	0.7
16	HLA-A*68:01	48	57	TTVSNMAEVR	0.2
17	HLA-A*68:02	47	56	TTTVSNMAEV	0.3
18	HLA-A*74:01	283	292	RLSSGHLKCR	0.8
**B Type**					
1	HLA-B*07:02	233	242	TPHWNNKEAL	0.35
2	HLA-B*08:01	298	307	LRLKGVSYSL	0.35
3	HLA-B*13:01	343	352	AQMAVDMQTL	0.2
4	HLA-B*13:02	41	50	VDIELVTTTV	0.6
5	HLA-B*14:02	415	424	VRGAKRMAVL	0.2
6	HLA-B*15:01	52	61	NMAEVRSYCY	0.45
7	HLA-B*15:02	81	90	YLDKQSDTQY	0.2
8	HLA-B*15:25	373	382	KMMLELDPPF	0.2
9	HLA-B*18:01	25	34	LEHGGCVTVM	0.8
10	HLA-B*27:02	298	307	LRLKGVSYSL	0.5
11	HLA-B*27:05	356	365	GRLITANPVI	0.65
12	HLA-B*35:01	305	314	YSLCTAAFTF	0.65
13	HLA-B*35:03	317	326	IPAETLHGTV	1.55

EXCLI Journal 2017;16:63-72 – ISSN 1611-2156
Received: October 20, 2016, accepted: January 02, 2017, published: January 13, 2017

S.No.	Allele	Start	End	Peptide/Epitopes	Percentile Rank
A Type	MHC-I				
14	HLA-B*37:01	319	328	AETLHGTVTV	0.3
15	HLA-B*38:01	398	407	HHWHRSGSTI	0.3
16	HLA-B*39:01	260	261	SQEGAVHTAL	0.2
17	HLA-B*40:01	275	284	AEMDGAKGRL	0.3
18	HLA-B*40:02	369	378	TENSKMMLEL	0.25
19	HLA-B*44:02	275	284	AEMDGAKGRL	0.25
20	HLA-B*44:03	275	284	AEMDGAKGRL	0.35
21	HLA-B*46:01	303	312	VSYSLCTAAF	0.2
22	HLA-B*48:01	343	352	AQMAVDMQTL	0.3
23	HLA-B*49:01	319	328	AETLHGTVTV	0.4
24	HLA-B*50:01	25	34	LEHGGCVTVM	0.4
25	HLA-B*51:01	380	389	PPFGDSYIVI	1.35
26	HLA-B*52:01	303	312	VSYSLCTAAF	0.5
27	HLA-B*53:01	11	20	FVEGMSGGTW	0.3
28	HLA-B*55:01	317	326	IPAETLHGTV	0.3
29	HLA-B*56:01	317	326	IPAETLHGTV	0.4
30	HLA-B*57:01	305	314	YSLCTAAFTF	2.2
31	HLA-B*58:01	420	429	RMAVLGDTAW	0.2
32	HLA-B*58:02	305	314	YSLCTAAFTF	0.3
C Type					
1	HLA-C*01:02	303	312	VSYSLCTAAF	0.5
2	HLA-C*02:02	305	314	YSLCTAAFTF	0.3
3	HLA-C*02:09	305	314	YSLCTAAFTF	0.3
4	HLA-C*03:02	305	314	YSLCTAAFTF	0.2
5	HLA-C*03:03	59	68	YCYEASISDM	0.2
6	HLA-C*03:04	305	314	YSLCTAAFTF	0.6
7	HLA-C*04:01	448	457	IFGAAFKSLF	0.3
8	HLA-C*05:01	81	90	YLDKQSDTQY	0.2
9	HLA-C*06:02	398	407	HHWHRSGSTI	0.8
10	HLA-C*07:01	248	257	AHAKRQTVVV	0.3
11	HLA-C*07:02	72	81	SRCPTQGEAY	0.9
12	HLA-C*07:04	298	307	LRLKGVSYSL	0.4
13	HLA-C*08:01	305	314	YSLCTAAFTF	0.5
14	HLA-C*08:02	382	391	FGDSYIVIGV	0.2

EXCLI Journal 2017;16:63-72 – ISSN 1611-2156
Received: October 20, 2016, accepted: January 02, 2017, published: January 13, 2017

S.No.	Allele	Start	End	Peptide/Epitopes	Percentile Rank
**C Type**					
15	HLA-C*12:02	305	314	YSLCTAAFTF	0.5
16	HLA-C*12:03	247	256	DAHAKRQTVV	0.5
17	HLA-C*14:02	313	322	TFTKIPAETL	1.0
18	HLA-C*15:02	367	376	ESTENSKMML	0.5
19	HLA-C*16:01	305	314	YSLCTAAFTF	0.2
20	HLA-C*17:01	346	355	AVDMQTLTPV	0.4
**E Type**					
1	HLA-E*01:01	466	475	ILIGTLLMWL	1.2
**G Type**					
1	HLA-G*01:01	305	314	YSLCTAAFTF	0.9
2	HLA-G*01:02	305	314	YSLCTAAFTF	0.9
3	HLA-G*01:03	305	314	YSLCTAAFTF	0.9
4	HLA-G*01:04	305	314	YSLCTAAFTF	0.9
5	HLA-G*01:06	305	314	YSLCTAAFTF	0.9

EXCLI Journal 2017;16:63-72 – ISSN 1611-2156
Received: October 20, 2016, accepted: January 02, 2017, published: January 13, 2017

Table S3: Immunogenicity assessment of the identified peptides using VaxiJen V2.0 tool

						Immunogenicity	
	**Type of Immunity and HLA Class**	**Start**	**End**	**Peptide**	**Percentile Rank**	**Threshold**	VAXIJEN Score
	**B-CELL**	194	213	TGLDFSDLYYLTMNN-KHWLV	0.90	0.4	1.2758 (Probable ANTIGEN)
	**T-CELL**						
	**HLA CLASS-I**						
**A Type**							
1	HLA- A*01:01	81	90	YLDKQSDTQY	0.25	0.4	0.2507 (Probable NON-ANTIGEN)
2	HLA-A*02:01	485	494	**SLMCLALGGV**	0.2	0.4	1.7363 (Probable ANTIGEN)
3	HLA-A*02:06	485	494	SLMCLALGGV	0.25	0.4	1.7363 (Probable ANTIGEN)
4	HLA-A*03:01	471	480	**LLMWLGLNTK**	0.25	0.4	1.8391 (Probable ANTIGEN)
5	HLA-A*11:01	114	123	VTCAKFACSK	0.45	0.4	-0.1024 (Probable NON-ANTIGEN)
6	HLA-A*23:01	448	457	IFGAAFKSLF	0.3	0.4	0.1395 (Probable NON-ANTIGEN)
7	HLA-A*24:02	448	457	IFGAAFKSLF	0.45	0.4	0.1395 (Probable NON-ANTIGEN)
8	HLA-A*25:01	177	186	**EATLGGFGSL**	1.7	0.4	1.0328 (Probable ANTIGEN)
9	HLA-A*26:01	377	386	**ELDPPFGDSY**	0.9	0.4	0.6953 (Probable ANTIGEN)
10	HLA-A*29:02	52	61	**NMAEVRSYCY**	0.3	0.4	1.2831 (Probable ANTIGEN)
11	HLA-A*30:01	409	418	**KAFEATVRGA**	1.35	0.4	0.9016 (Probable ANTIGEN)
12	HLA-A*30:02	193	202	**RTGLDFSDLY**	0.25	0.4	1.8346 (Probable ANTIGEN)
13	HLA-A*31:01	283	292	**RLSSGHLKCR**	0.7	0.4	2.0901 (Probable ANTIGEN
14	HLA-A*32:01	305	314	YSLCTAAFTF	1.0	0.4	0.1343 (Probable NON-ANTIGEN)
15	HLA-A*33:03	348	357	**DMQTLTPVGR**	0.7	0.4	0.8543 (Probable ANTIGEN)
16	HLA-A*68:01	48	57	**TTVSNMAEVR**	0.2	0.4	0.7175 (Probable ANTIGEN)
17	HLA-A*68:02	47	56	**TTTVSNMAEV**	0.3	0.4	0.6972 (Probable ANTIGEN)
18	HLA-A*74:01	283	292	RLSSGHLKCR	0.8	0.4	2.0901 (Probable ANTIGEN)
**B Type**							
1	HLA-B*07:02	233	242	**TPHWNNKEAL**	0.35	0.4	1.5710 (Probable ANTIGEN)
2	HLA-B*08:01	298	307	**LRLKGVSYSL**	0.35	0.4	1.1682 (Probable ANTIGEN)
3	HLA-B*13:01	343	352	AQMAVDMQTL	0.2	0.4	0.3205 (Probable NON-ANTIGEN)
4	HLA-B*13:02	41	50	**VDIELVTTTV**	0.6	0.4	1.3702 (Probable ANTIGEN)
5	HLA-B*14:02	415	424	VRGAKRMAVL	0.2	0.4	0.2533 (Probable NON-ANTIGEN)
6	HLA-B*15:01	52	61	NMAEVRSYCY	0.45	0.4	1.2831 (Probable ANTIGEN)

						Immunogenicity	
	**Type of Immunity and HLA Class**	**Start**	**End**	**Peptide**	**Percentile Rank**	**Threshold**	VAXIJEN Score
	**HLA CLASS-I**						
7	HLA-B*15:02	81	90	YLDKQSDTQY	0.2	0.4	0.2507 (Probable NON-ANTIGEN)
8	HLA-B*15:25	373	382	**KMMLELDPPF**	0.2	0.4	1.0318 (Probable ANTIGEN)
9	HLA-B*18:01	25	34	LEHGGCVTVM	0.8	0.4	0.3115 (Probable NON-ANTIGEN)
10	HLA-B*27:02	298	307	LRLKGVSYSL	0.5	0.4	1.1682 (Probable ANTIGEN)
11	HLA-B*27:05	356	365	GRLITANPVI	0.65	0.4	-0.2339 (Probable NON-ANTIGEN)
12	HLA-B*35:01	305	314	YSLCTAAFTF	0.65	0.4	0.1343 (Probable NON-ANTIGEN)
13	HLA-B*35:03	317	326	IPAETLHGTV	1.55	0.4	-0.0361 (Probable NON-ANTIGEN)
14	HLA-B*37:01	319	328	**AETLHGTVTV**	0.3	0.4	0.5422 (Probable ANTIGEN)
15	HLA-B*38:01	398	407	**HHWHRSGSTI**	0.3	0.4	0.4776 (Probable ANTIGEN)
16	HLA-B*39:01	260	261	SQEGAVHTAL	0.2	0.4	0.0330 (Probable NON-ANTIGEN)
17	HLA-B*40:01	275	284	AEMDGAKGRL	0.3	0.4	0.2032 (Probable NON-ANTIGEN)
18	HLA-B*40:02	369	378	TENSKMMLEL	0.25	0.4	0.3817 (Probable NON-ANTIGEN)
19	HLA-B*44:02	275	284	AEMDGAKGRL	0.25	0.4	0.2032 (Probable NON-ANTIGEN)
20	HLA-B*44:03	275	284	AEMDGAKGRL	0.35	0.4	0.2032 (Probable NON-ANTIGEN)
21	HLA-B*46:01	303	312	VSYSLCTAAF	0.2	0.4	-0.1391 (Probable NON-ANTIGEN)
22	HLA-B*48:01	343	352	AQMAVDMQTL	0.3	0.4	0.3205 (Probable NON-ANTIGEN)
23	HLA-B*49:01	319	328	AETLHGTVTV	0.4	0.4	0.5422 (Probable ANTIGEN)
24	HLA-B*50:01	25	34	LEHGGCVTVM	0.4	0.4	0.3115 (Probable NON-ANTIGEN)
25	HLA-B*51:01	380	389	PPFGDSYIVI	1.35	0.4	0.2388 (Probable NON-ANTIGEN)
26	HLA-B*52:01	303	312	VSYSLCTAAF	0.5	0.4	-0.1391 (Probable NON-ANTIGEN)
27	HLA-B*53:01	11	20	FVEGMSGGTW	0.3	0.4	-0.0469 (Probable NON-ANTIGEN)
28	HLA-B*55:01	317	326	IPAETLHGTV	0.3	0.4	-0.0361 (Probable NON-ANTIGEN)
29	HLA-B*56:01	317	326	IPAETLHGTV	0.4	0.4	-0.0361 (Probable NON-ANTIGEN)
30	HLA-B*57:01	305	314	YSLCTAAFTF	2.2	0.4	0.1343 (Probable NON-ANTIGEN)
31	HLA-B*58:01	420	429	**RMAVLGDTAW**	0.2	0.4	0.8599 (Probable ANTIGEN)
32	HLA-B*58:02	305	314	YSLCTAAFTF	0.3	0.4	0.1343 (Probable NON-ANTIGEN)

						Immunogenicity	
	**Type of Immunity and HLA Class**	**Start**	**End**	**Peptide**	**Percentile Rank**	**Threshold**	VAXIJEN Score
	**HLA CLASS-I**						
**C Type**							
1	HLA-C*01:02	303	312	VSYSLCTAAF	0.5	0.4	-0.1391 (Probable NON-ANTIGEN)
2	HLA-C*02:02	305	314	YSLCTAAFTF	0.3	0.4	0.1343 (Probable NON-ANTIGEN)
3	HLA-C*02:09	305	314	YSLCTAAFTF	0.3	0.4	0.1343 (Probable NON-ANTIGEN)
4	HLA-C*03:02	305	314	YSLCTAAFTF	0.2	0.4	0.1343 (Probable NON-ANTIGEN)
5	HLA-C*03:03	59	68	**YCYEASISDM**	0.2	0.4	1.0191 (Probable ANTIGEN)
6	HLA-C*03:04	305	314	YSLCTAAFTF	0.6	0.4	0.1343 (Probable NON-ANTIGEN)
7	HLA-C*04:01	448	457	IFGAAFKSLF	0.3	0.4	0.1395 (Probable NON-ANTIGEN)
8	HLA-C*05:01	81	90	YLDKQSDTQY	0.2	0.4	0.2507 (Probable NON-ANTIGEN)
9	HLA-C*06:02	398	407	HHWHRSGSTI	0.8	0.4	0.4776 (Probable ANTIGEN)
10	HLA-C*07:01	248	257	**AHAKRQTVVV**	0.3	0.4	0.7216 (Probable ANTIGEN)
11	HLA-C*07:02	72	81	**SRCPTQGEAY**	0.9	0.4	0.6146 (Probable ANTIGEN)
12	HLA-C*07:04	298	307	LRLKGVSYSL	0.4	0.4	1.1682 (Probable ANTIGEN)
13	HLA-C*08:01	305	314	YSLCTAAFTF	0.5	0.4	0.1343 (Probable NON-ANTIGEN)
14	HLA-C*08:02	382	391	**FGDSYIVIGV**	0.2	0.4	0.6413 (Probable ANTIGEN)
15	HLA-C*12:02	305	314	YSLCTAAFTF	0.5	0.4	0.1343 (Probable NON-ANTIGEN)
16	HLA-C*12:03	247	256	**DAHAKRQTVV**	0.5	0.4	0.6538 (Probable ANTIGEN)
17	HLA-C*14:02	313	322	TFTKIPAETL	1.0	0.4	-0.6223 (Probable NON-ANTIGEN)
18	HLA-C*15:02	367	376	**ESTENSKMML**	0.5	0.4	0.4843 (Probable ANTIGEN)
19	HLA-C*16:01	305	314	YSLCTAAFTF	0.2	0.4	0.1343 (Probable NON-ANTIGEN)
20	HLA-C*17:01	346	355	**AVDMQTLTPV**	0.4	0.4	0.7836 (Probable ANTIGEN)
**E Type**							
1	HLA-E*01:01	466	475	ILIGTLLMWL	1.2	0.4	0.0776 (Probable NON-ANTIGEN)
**G Type**							
1	HLA-G*01:01	305	314	YSLCTAAFTF	0.9	0.4	0.1343 (Probable NON-ANTIGEN)
2	HLA-G*01:02	305	314	YSLCTAAFTF	0.9	0.4	0.1343 (Probable NON-ANTIGEN)
3	HLA-G*01:03	305	314	YSLCTAAFTF	0.9	0.4	0.1343 (Probable NON-ANTIGEN)
4	HLA-G*01:04	305	314	YSLCTAAFTF	0.9	0.4	0.1343 (Probable NON-ANTIGEN)
5	HLA-G*01:06	305	314	YSLCTAAFTF	0.9	0.4	0.1343 (Probable NON-ANTIGEN)

EXCLI Journal 2017;16:63-72 – ISSN 1611-2156
Received: October 20, 2016, accepted: January 02, 2017, published: January 13, 2017

						**Immunogenicity**	
	**Type of Immunity and HLA Class**	**Start**	**End**	**Peptide**	**Percentile Rank**	**Threshold**	VAXIJEN Score
	**HLA CLASS-II**						
**DP Type**							
1	HLA-DPA1*01/DPB1*04:01	193	207	**RTGLDFSDLYYLTMN**	0.61	0.4	1.4016 (Probable ANTIGEN)
2	HLA-DPA1*01:03/DPB1*02:01	192	206	**PRTGLDFSDLYYLTM**	0.05	0.4	1.4164 (Probable ANTIGEN)
3	HLA-DPA1*02:01/DPB1*01:01	460	474	MSWFSQILIGTLLMW	1.54	0.4	0.1571 (Probable NON-ANTIGEN)
4	HLA-DPA1*02:01/DPB1*05:01	443	457	KGIHQIFGAAFKSLF	6.57	0.4	-0.1597 (Probable NON-ANTIGEN)
5	HLA-DPA1*03:01/DPB1*04:02	462	476	WFSQILIGTLLMWLG	1.1	0.4	0.1606 (Probable NON-ANTIGEN)
**DQ Type**							
1	HLA-DQA1*01:01/DQB1*05:01	211	225	**WLVHKEWFHDIPLPW**	0.52	0.4	0.6855 (Probable ANTIGEN)
2	HLA-DQA1*01:02/DQB1*06:02	478	492	**NTKNGSISLMCLALG**	1.15	0.4	1.9904 (Probable ANTIGEN)
3	HLA-DQA1*03:01/DQB1*03:02	264	278	**AVHTALAGALEAEMD**	0.79	0.4	0.4323 (Probable ANTIGEN)
4	HLA-DQA1*04:01/DQB1*04:02	264	278	AVHTALAGALEAEMD	0.64	0.4	0.4323 (Probable ANTIGEN)
5	HLA-DQA1*05:01/DQB1*02:01	264	278	AVHTALAGALEAEMD	2.16	0.4	0.4323 (Probable ANTIGEN)
6	HLA-DQA1*05:01/DQB1*03:01	262	276	**EGAVHTALAGALEAE**	0.93	0.4	0.4632 (Probable ANTIGEN)

EXCLI Journal 2017;16:63-72 – ISSN 1611-2156
Received: October 20, 2016, accepted: January 02, 2017, published: January 13, 2017

						Immunogenicity	
	**Type of Immunity and HLA Class**	**Start**	**End**	**Peptide**	**Percentile Rank**	**Threshold**	VAXIJEN Score
	**HLA CLASS-II**						
**DR Type**							
1	HLA-DRB1*01:01	309	323	TAAFTFTKIPAETLH	1.58	0.4	0.3401 (Probable NON-ANTIGEN)
2	HLA-DRB1*01:02	131	145	**QPENLEYRIMLSVHG**	0.23	0.4	0.4589 (Probable ANTIGEN)
3	HLA-DRB1*03:01	191	205	**EPRTGLDFSDLYYLT**	0.72	0.4	1.1568 (Probable ANTIGEN)
4	HLA-DRB1*03:05	292	306	**RLKMDKLRLKGVSYS**	0.67	0.4	0.7441 (Probable ANTIGEN)
5	HLA-DRB1*03:06	469	483	**GTLLMWLGLNTKNGS**	0.18	0.4	1.2662 (Probable ANTIGEN)
6	HLA-DRB1*03:07	469	483	GTLLMWLGLNTKNGS	0.18	0.4	1.2662 (Probable ANTIGEN
7	HLA-DRB1*03:08	469	483	GTLLMWLGLNTKNGS	0.18	0.4	1.2662 (Probable ANTIGEN)
8	HLA-DRB1*03:11	489	303	LKCRLKMDKLRLKGV	0.05	0.4	-0.0874 (Probable NON-ANTIGEN)
9	HLA-DRB1*04:01	197	211	**DFSDLYYLTMNNKHW**	0.11	0.4	1.1408 (Probable ANTIGEN)
10	HLA-DRB1*04:02	292	306	RLKMDKLRLKGVSYS	0.17	0.4	0.7441 (Probable ANTIGEN)
11	HLA-DRB1*04:04	489	503	**LALGGVLIFLSTAVS**	0.3	0.4	0.4505 (Probable ANTIGEN)
12	HLA-DRB1*04:05	198	212	**FSDLYYLTMNNKHWL**	0.3	0.4	0.8870 (Probable ANTIGEN)
13	HLA-DRB1*04:08	131	145	QPENLEYRIMLSVHG	0.03	0.4	0.4589 (Probable ANTIGEN)
14	HLA-DRB1*04:10	131	145	QPENLEYRIMLSVHG	0.07	0.4	0.4589 (Probable ANTIGEN)
15	HLA-DRB1*04:21	131	145	QPENLEYRIMLSVHG	0.02	0.4	0.4589 (Probable ANTIGEN)
16	HLA-DRB1*04:23	489	503	LALGGVLIFLSTAVS	0.03	0.4	0.4505 (Probable ANTIGEN)
17	HLA-DRB1*04:26	131	145	QPENLEYRIMLSVHG	0.09	0.4	0.4589 (Probable ANTIGEN)
18	HLA-DRB1*07:01	490	504	ALGGVLIFLSTAVSA	0.71	0.4	0.3846 (Probable NON-ANTIGEN)
19	HLA-DRB1*07:03	174	188	**PRAEATLGGFGSLGL**	0.58	0.4	1.4679 (Probable ANTIGEN)
20	HLA-DRB1*08:01	84	98	KQSDTQYVCKRTLVD	0.01	0.4	0.0137 (Probable NON-ANTIGEN)
21	HLA-DRB1*08:02	489	503	LALGGVLIFLSTAVS	0.42	0.4	0.4505 (Probable ANTIGEN)
22	HLA-DRB1*08:04	292	306	RLKMDKLRLKGVSYS	0.01	0.4	0.7441 (Probable ANTIGEN)
23	HLA-DRB1*08:06	292	306	RLKMDKLRLKGVSYS	0.03	0.4	0.7441 (Probable ANTIGEN)
24	HLA-DRB1*08:13	292	306	RLKMDKLRLKGVSYS	0.01	0.4	0.7441 (Probable ANTIGEN)
25	HLA-DRB1*09:01	308	322	CTAAFTFTKIPAETL	0.46	0.4	0.1799 (Probable NON-ANTIGEN)
26	HLA-DRB1*11:01	489	503	LALGGVLIFLSTAVS	0.34	0.4	0.4505 (Probable ANTIGEN)
27	HLA-DRB1*11:02	292	306	RLKMDKLRLKGVSYS	0.02	0.4	0.7441 (Probable ANTIGEN)
28	HLA-DRB1*11:04	489	503	LALGGVLIFLSTAVS	0.16	0.4	0.4505 (Probable ANTIGEN)
29	HLA-DRB1*11:06	489	503	LALGGVLIFLSTAVS	0.16	0.4	0.4505 (Probable ANTIGEN)

EXCLI Journal 2017;16:63-72 – ISSN 1611-2156
Received: October 20, 2016, accepted: January 02, 2017, published: January 13, 2017

						Immunogenicity	
	**Type of Immunity and HLA Class**	**Start**	**End**	**Peptide**	**Percentile Rank**	**Threshold**	VAXIJEN Score
	**HLA CLASS-II**						
**DR Type**							
30	HLA-DRB1*11:07	292	306	RLKMDKLRLKGVSYS	0.29	0.4	0.7441 (Probable ANTIGEN)
31	HLA-DRB1*11:14	292	306	RLKMDKLRLKGVSYS	0.09	0.4	0.7441 (Probable ANTIGEN)
32	HLA-DRB1*11:20	131	145	QPENLEYRIMLSVHG	0.08	0.4	0.4589 (Probable ANTIGEN)
33	HLA-DRB1*11:21	292	306	RLKMDKLRLKGVSYS	0.02	0.4	0.7441 (Probable ANTIGEN)
34	HLA-DRB1*11:28	131	145	QPENLEYRIMLSVHG	0.25	0.4	0.4589 (Probable ANTIGEN)
35	HLA-DRB1*12:01	490	504	ALGGVLIFLSTAVSA	0.9	0.4	0.3846 (Probable NON-ANTIGEN)
36	HLA-DRB1*13:01	292	306	RLKMDKLRLKGVSYS	0.04	0.4	0.7441 (Probable ANTIGEN)
37	HLA-DRB1*13:02	354	368	PVGRLITANPVITES	3.21	0.4	-0.0285 (Probable NON-ANTIGEN)
38	HLA-DRB1*13:04	292	306	RLKMDKLRLKGVSYS	0.15	0.4	0.7441 (Probable ANTIGEN)
39	HLA-DRB1*13:05	131	145	QPENLEYRIMLSVHG	0.25	0.4	0.4589 (Probable ANTIGEN)
40	HLA-DRB1*13:07	489	503	LALGGVLIFLSTAVS	0.06	0.4	0.4505 (Probable ANTIGEN)
41	HLA-DRB1*13:11	489	503	LALGGVLIFLSTAVS	0.16	0.4	0.4505 (Probable ANTIGEN)
42	HLA-DRB1*13:21	84	98	KQSDTQYVCKRTLVD	0.15	0.4	0.0137 (Probable NON-ANTIGEN)
43	HLA-DRB1*13:22	292	306	RLKMDKLRLKGVSYS	0.02	0.4	0.7441 (Probable ANTIGEN)
44	HLA-DRB1*13:23	292	306	RLKMDKLRLKGVSYS	0.09	0.4	0.7441 (Probable ANTIGEN)
45	HLA-DRB1*13:27	292	306	RLKMDKLRLKGVSYS	0.04	0.4	0.7441 (Probable ANTIGEN)
46	HLA-DRB1*15:01	488	502	CLALGGVLIFLSTAV	0.07	0.4	0.5558 (Probable ANTIGEN)
47	HLA-DRB1*15:02	174	188	PRAEATLGGFGSLGL	0.02	0.4	1.4679 (Probable ANTIGEN)
48	HLA-DRB1*15:06	174	188	PRAEATLGGFGSLGL	0.06	0.4	1.4679 (Probable ANTIGEN)
49	HLA-DRB3*01:01	370	384	ENSKMMLELDPPFGD	0.01	0.4	0.7744 (Probable ANTIGEN)
50	HLA-DRB4*01:01	290	304	KCRLKMDKLRLKGVS	2.76	0.4	-0.0023 (Probable NON-ANTIGEN)
51	HLA-DRB5*01:01	450	464	GAAFKSLFGGMSWFS	0.31	0.4	0.2836 (Probable NON-ANTIGEN)
52	HLA-DRB5*01:05	131	145	QPENLEYRIMLSVHG	0.17	0.4	0.4589 (Probable ANTIGEN)
